# Establishing semantic relatedness through ratings, reaction times, and semantic vectors: A database in Polish

**DOI:** 10.1371/journal.pone.0284801

**Published:** 2023-04-24

**Authors:** Karolina Rataj, Patrycja Kakuba, Paweł Mandera, Walter J. B. van Heuven

**Affiliations:** 1 Faculty of English, Neuroscience of Language Laboratory, Adam Mickiewicz University, Poznań, Poland; 2 Faculty of English, Department of Psycholinguistic Studies, Adam Mickiewicz University, Poznań, Poland; 3 Lingvist Technologies, Tallinn, Estonia; 4 School of Psychology, University of Nottingham, Nottingham, United Kingdom; Centre National de la Recherche Scientifique, FRANCE

## Abstract

This study presents a Polish semantic priming dataset and semantic similarity ratings for word pairs obtained with native Polish speakers, as well as a range of semantic spaces. The word pairs include strongly related, weakly related, and semantically unrelated word pairs. The rating study (Experiment 1) confirmed that the three conditions differed in semantic relatedness. The semantic priming lexical decision study with a carefully matched subset of the stimuli (Experiment 2), revealed strong semantic priming effects for strongly related word pairs, whereas weakly related word pairs showed a smaller but still significant priming effect relative to semantically unrelated word pairs. The datasets of both experiments and those of SimLex-999 for Polish were then used in a robust semantic model selection from existing and newly trained semantic spaces. This database of semantic vectors, semantic relatedness ratings, and behavioral data collected for all word pairs enable future researchers to benchmark new vectors against this dataset. Furthermore, the new vectors are made freely available for researchers. Although similar semantically strongly and weakly related word pairs are available in other languages, this is the first freely available database for Polish, that combines measures of semantic distance and human data.

## Introduction

Semantic relatedness can be understood as any type of a semantic relationship between two concepts or words [1]. This relationship has often been examined in behavioral studies, in which participants are presented with semantically related, e.g., *butcher* and *ham*, and unrelated, e.g., *nail* and *ham*, pairs of words. Results of such studies have revealed that reaction times (RTs) in lexical decision tasks to target words preceded by semantically related word primes are faster than when preceded by unrelated word primes, a phenomenon known as semantic priming.

### Semantic relatedness and semantic priming

When semantic relatedness is treated as a binary phenomenon with only close semantic relations and items that are semantically unrelated, the complexity of semantic relatedness remains concealed [2,3]. To address this issue, research has also used indirect semantic relations, which have been defined as associations that can only be made if an intermediate link between the two words can be identified. For example, *lemon* can be associated with *sweet* through the indirect association between *lemon-sour* and *sour-sweet* [2], or *lion* can be associated with *stripes* through the indirect association between *lion-tiger* and *tiger-stripes* [4–6].

Several studies that used the intermediate category of weakly related word pairs have shown a graded priming effect, with close relations evoking shortest RTs, unrelated items longest RTs, and RTs for weakly related items falling in-between, suggesting an underlying linear relationship [2,7]. It is obvious that indirect semantic relations do not exhaust all possible remote semantic relations between words. For some word pairs, deriving meaning, or finding a semantic relationship between two words does not require this intermediate step. It might involve finding direct, but distant semantic links between words that are weakly related. The word pair *decoration—brick* can represent such a remote relationship (compared to a strongly related word pair *wall*—*brick*), for which this intermediate step is not required.

Ortu, Allan [8] examined such relations in an event-related potential (ERP) study, with word pairs representing moderate and high degrees of association. Their behavioral data showed a similar linear pattern as the one observed in studies focusing on direct and indirect relations, with RTs for moderately associated word pairs falling in-between those for highly associated and unrelated items. The strength of the associations used in this study was established through word association databases. However, such sources do not exist for many languages, including Polish. For these languages, materials for semantic priming studies with items varying in the strength of the semantic relationship require conducting rating studies, in which human participants evaluate the items on a scale ranging from *very related in meaning* to *not related at all*. However, recently, distributional semantics have been utilized by researchers to select items based on their semantic distance in a semantic space [9–11].

Some studies have pointed out a possible difference between semantic and associative relations [1,8]. These studies defined semantic relations using feature overlap (e.g., *mouse* and *rat*) or linguistic interchangeability (e.g., *cup* and *mug*), whereas associative relations were defined based on the frequency of co-occurrence in linguistic contexts. Semantic priming effects have been observed for both types of semantic relations [12,13]. Nevertheless, the ongoing discussion about the distinction between semantic and associative relations emphasizes that current experimental paradigms face a challenge in reaching clear conclusions [14,15] because it is difficult to establish whether feature overlap or association strength accounts for observed semantic/associative priming effects, due to the potential involvement of both. It is beyond the scope of this paper to address this issue, for which reason we refer in this paper to *semantic relatedness*.

Whether, and to what extent, two words are semantically related has frequently been established through normative studies conducted prior to behavioral experiments. Commonly, these are rating studies in which participants decide whether or how much two words are related in meaning [12,16–18]. To identify the underlying continuum of semantic relations, a Likert scale rather than a binary decision task has been used. Individual semantic similarity judgements employed to determine semantic relatedness are, however, time-consuming, require involvement of a large number of participants, and can potentially involve several issues, including bias or survey fatigue, that can impact the judgements [19,20].

Another way to obtain information about semantic relatedness between words is to use large-scale word association databases. These are constructed on the basis of word association tasks performed by a large number of participants [21–23]. In the word association task, participants are presented with a trigger word and asked to generate one, two or three words that first come to mind [16,17,21–23]. The advantage of using such databases is that once they have been created, they are a large source of easily available data for stimulus selection. However, the limitation is that most of these databases are developed for English, and, as far as we are aware, there is no word association database for Polish.

Semantic priming effects have been used as a more sensitive measure of the strength of semantic relations [8,12,16,24]. Such effects have been observed in both lexical decision tasks (LDT), in which participants decide whether a word presented on the screen is or is not an existing word in a given language [12,16,17], and in semantic judgment tasks (SJT), in which participants decide whether or not two words are related in meaning [8]. The advantage of the LDT is that participants do not focus on the semantic relationship as such, and results of these studies are assumed to point to the automatic nature of semantic priming effects. In the SJT, which has been used less frequently in the literature, participants explicitly evaluate the semantic relationship between the two words. A comparison of research results suggests that the task does not play a crucial role in the general pattern of semantic priming effects, i.e., in both tasks directly related prime-target words pairs show facilitation in comparison to unrelated word pairs [2,7].

### Relatedness measure from distributional semantics

Because collecting semantic relatedness measures from human subjects is time consuming and potentially costly, researchers have used large text corpora to create semantic spaces that can be used to obtain estimates of semantic relatedness of word pairs [9,10]. This distributional semantics approach is based on the idea that the patterns in which words occur in text corpora can be used to infer information about semantic relatedness [25,26]. One of the frequently applied methods to obtain such measures is *word2vec* [27], which involves training a 3-layer neural network (input, hidden, and output layers) to predict a target word from the context words (continuous bag of words model or cbow) or the context words from the target word (skipgram model). After training, word vectors are obtained from the neural network model by taking the weights of each word to the hidden layer in the network. The distance in the multidimensional vector space between two words is then considered to be a proxy measure of the semantic relatedness (the closer the two words in the space the more related they are; for an explanation of the theoretical underpinnings of representing words as points in multidimensional space see [26,28]).

There are various ways to train the model to create a semantic space as different corpora and hyperparameters can produce vastly different results. It is frequently reported that different semantic spaces often turn out to be optimal for predicting human behavior in different tasks [10,11]. Ideally researchers would like to know which semantic space they can rely on, for example when selecting stimuli for their experiments. This means that, in practice, it is desirable to be able to select “the best model”. To do that, similarly to word frequency norm selection studies [29–32], a pragmatic definition of model quality can be adopted: the model that predicts some behavioral measure(s) better than another model can be considered to have higher quality. In the case of semantic spaces, a space that better predicts semantic priming LDT RTs and human relatedness or similarity ratings than another space can be considered better. Ideally, predictions would also generalize well across tasks and stimuli sets.

The procedure of selecting an optimal space based on its performance in predicting behavioral data is quite robust when large behavioral datasets [24,33,34] are used for evaluation and model selection. However, such datasets do not exist for most languages, including Polish, and much smaller ones have to be used. This increases the chance that the result will be distorted due to overfitting originating from training many models with different hyperparameters or some other alterations in the training procedure. When overfitting happens, the predictiveness of the model on an independent dataset is also likely to be overestimated.

To illustrate how such a distortion may occur in practice, we propose the following thought experiment. The hyperparameters of the model and the corpus used influence the quality of the model. Imagine an extreme case in which there is a configuration of hyperparameters that produces a semantic model that is completely random. Such a model would clearly have a very low quality. At the same time if enough of such models are “trained”, purely by chance one of them could produce a set of predictions that allow to predict (correlate with) some behavioral measure well. This could mislead a researcher into believing that such a low-quality semantic space is actually a well-performing one, even though it would likely not perform well on any new dataset. Instead, the desirable outcome of the semantic space selection involves identifying a model that is likely to generalize well also to new datasets and correctly estimate how good the model is when applied to independent datasets.

### The present study

In the present study, we used both human data and semantic vectors to establish the strength of semantic relations for a selected set of word pairs in Polish, with the aim of comparing the predictive power of semantic priming effects, human ratings, and semantic distances obtained from a large number of semantic spaces. We trained semantic spaces using a wide range of hyperparameter settings to demonstrate that such an overfitting can occur in practice with smaller datasets and propose an approach that helps to remedy this problem. Our approach is based on reducing the possible overestimation of the model performance on independent datasets by using nested cross-validation–a method that is frequently applied in machine learning research to deal with problems with model performance estimation when multiple models with different hyperparameter sets have to be considered [35].

In addition, we aimed at reducing the overfitting problem by jointly considering the performance of the models on multiple datasets. In contrast to other studies that evaluated the performance of different semantic spaces independently on each dataset [10,36], we tried to consider various types of behavioral data simultaneously. The idea behind this procedure is to identify a model that finds a compromise between performance on different tasks, and hence can be expected to generalize better. In addition, this approach allows to compute the performance of the models on an aggregation of multiple datasets, which, to some extent, minimizes potential problems related to their individual small sizes.

To summarize, in this paper we combine multiple approaches with the aim of developing a well-matched set of materials for studies on semantic relatedness, in which, in addition to strongly related and unrelated items, weakly related word pairs are included. These weakly related pairs are of particular interest for research on semantic relations, semantic processing, meaning construction, or creativity. We developed the materials in Polish, a language that has for a long time been underrepresented in research on semantic relations using human data and semantic spaces.

The first step in the development of the materials involved selecting word pairs based on their semantic distance in a semantic space [11]. Next, two judges selected the best pairs representing close and remote relations based on semantic distance. Additionally, unrelated word pairs were created and semantic distances confirmed that these word pairs were unrelated. The selected sample of 264 word pairs in each of the three conditions (strongly related, weakly related, and unrelated) was assessed in a rating study (Experiment 1). Based on the results of this study, a final set of 216 word pairs in three conditions was examined in a reaction time study, which employed a semantic priming paradigm with the LDT (Experiment 2). We employed the LDT, as this task has been widely used to examine the automatic nature of semantic processing. Finally, the set of semantic spaces published by Mykowiecka, Marciniak [11], as well as a newly trained set of semantic spaces, which covered a wide range of hyperparameters, were tested to obtain the best model that could be used by other researchers and to propose a robust method of semantic model selection that can be used for other languages.

## Experiment 1: Semantic relatedness ratings

The aim of this study was to obtain semantic relatedness ratings for Polish word pairs across three different semantic relatedness conditions: strongly related, weakly related, and unrelated. Semantic vectors were used to select 264 word pairs per condition. Because semantic vectors are not informative in terms of symmetry of the semantic relations (forward vs. backward associates), two rating surveys were conducted that differed in the order in which the two words in each word pair were presented, to ensure that ratings are not affected by the order of the word presentation.

### Methods

#### Participants

In both surveys, participants completed all five blocks (see Procedure). In Survey 1, 41 participants received links to the five blocks and were asked to complete one block a day for five consecutive days. Twelve participants filled out at least one block, but did not complete the study. The data from these participants were excluded from the analyses. Twenty-nine participants filled out all five blocks (10 males; *M*_age_ = 30.03, *SD* = 10.6).

In survey 2, participants were first year students in the bachelor program at the Faculty of English, Adam Mickiewicz University in Poznań. Out of 44 participants who volunteered to participate in the study, 37 participants completed all blocks (8 males; *M*_age_ = 19.7, *SD* = 0.9). Previous studies have used data from both below [16,17] or above [18] 20 participants per item. We decided to have data of at least 25 participants in each survey.

#### Materials

A total of 264 target words were selected from SUBTLEX-PL (Mandera et al. 2015). All target words were singular concrete nouns in the nominative case. The target words did not include any abstract nouns, compound nouns, proper names, Polish-English cognates, or interlingual homographs. Prime and target word characteristics are presented in Table 1.

**Table 1:** Prime and target word characteristics of 264 word pair triplets: Length, word frequency (Zipf values [30]), and the semantic relatedness values (cosine similarity scores) for the strongly related, weakly related, and unrelated conditions tested in Survey 1 and Survey 2.

Characteristics	Mean	SD (min.—max.)
Target word frequency (Zipf)	3.6	0.4 (2.9–4.7)
Target word length	6.3	1.4 (4–9)
***Prime word frequency (Zipf)***		
strongly related	2.9	0.8 (1.1–5.5)
weakly related	3.2	0.8 (1.3–5.3)
unrelated	3.5	0.4 (2.9–4.7)
***Prime word length***		
strongly related	7.1	2.2 (3–15)
weakly related	6.4	1.7 (3–12)
unrelated	6.7	1.4 (4–9)
***Semantic relatedness values***		
strongly related	0.69	.07 (0.52–0.87)
weakly related	0.49	.04 (0.41–0.59)
unrelated	0.12	.13 (-0.18–0.56)

For each target word, a strongly and weakly semantically related prime word was chosen using semantic vectors created from a large Polish corpus (nkjp+wiki-forms-all-300-cbow-ns, http://dsmodels.nlp.ipipan.waw.pl) created for the CoDeS project (http://zil.ipipan.waw.pl/CoDeS). First, 800 words with the highest cosine similarity scores with each target word were selected using the semantic vectors and the gensim Python library (https://radimrehurek.com/gensim/). Next, two human judges selected prime words from this list for the strongly related and weakly related conditions. Finally, prime words for the unrelated condition were selected from SUBTLEX-PL [30] using the same criteria as for the target words. Cosine similarity scores for these unrelated word pairs were also calculated. Examples of prime-target word pairs are included in Table 2.

**Table 2:** Examples of strongly related, weakly related and unrelated prime words together with target words in Polish selected for Survey 1 and Survey 2 (translations in English are provided in parentheses).

Strongly related	Weakly related	Unrelated	Target word
nadzienie (*filling*)	ziarno (*grain*)	grobowiec (*tomb*)	ciasto (*cake*)
spiżarnia (*pantry*)	zapiekanka (*casserole*)	ustawa (*act*)	kuchnia (*kitchen*)
filiżanka (*teacup*)	misa (*bowl*)	wojownik (*warrior*)	łyżka (*spoon*)

#### Design

The 264 word dyads in three conditions (strongly related, weakly related and unrelated) were tested in two surveys. The prime-target pairs were counterbalanced to avoid repetition of the target words in each block and divided into 5 short blocks. Four of the blocks contained 162 word pairs (54 target-prime pairs in each condition) and one block contained 144 word pairs (48 target-prime pairs in each condition). Only one target word occurred twice, once as a target word in block 2 and once as a prime word in block 4, all other target words only occurred once in the survey. Thirty prime words occurred twice, but only in four cases a prime word was repeated within the same block. In both surveys all word pairs were presented. The difference between the two surveys was that in Survey 1 the target preceded the prime word and in Survey 2 the prime preceded the target word.

#### Procedure

Each participant completed all five blocks, one at a time in one session across five days. The procedure was spread over several days because we wanted the same participant to see all the word pairs. In this within-subject design we found it crucial to ensure that the participants did not evaluate the same target word in three different conditions within one session (one day) to avoid any influence of repetition of target words on these evaluations.

The participants evaluated each word pair on a scale from 1 *bardzo niepowiązane* (*very unrelated)* to 7 *bardzo powiązane* (*very related)*, with the middle point 4 *nie mam zdania* (*I have no opinion)*. Each survey began with demographic questions. In both surveys, the order of word pairs was randomized, and the order of blocks was counterbalanced across participants.

Both surveys were administered using SurveyMonkey (https://www.surveymonkey.com/). Survey 1 was conducted online. Survey 2 was conducted at the Faculty of English in the Language and Communication Laboratory. The participants completed one block daily with a maximum timespan of three days between blocks. In both surveys, participants were presented with a description of the study and a consent form on the computer screen. If they agreed to participate (by pressing an *agree* button), the survey started. If they disagreed, the *Thank you* page was displayed. The study was approved by the Ethics Committee for Research Involving Human Participants at Adam Mickiewicz University.

### Results

**Fig 1:**
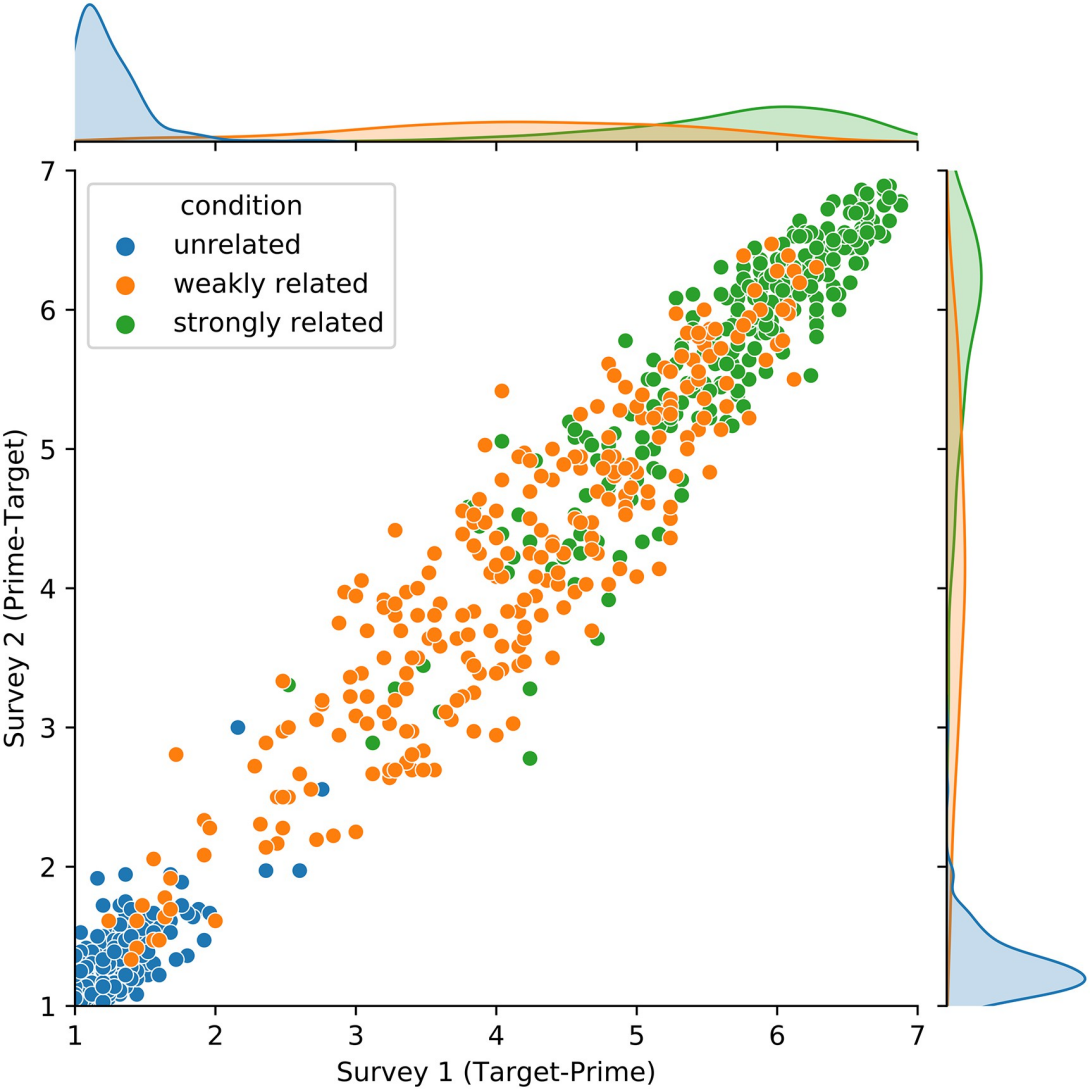
Scatterplot of the rating data from Survey 1 and 2.

On the basis of z-scores higher or lower than 2.5 SD, four participants were identified as outliers and excluded from the analysis in Survey 1 and one participant in Survey 2. Thus, the analyses were performed on the data from 25 participants in Survey 1 and 36 participants in Survey 2. Mean ratings of the remaining participant data of Survey 1 and 2 are presented in Table 3 and the combined data are presented in Fig 1. Pearson correlation between rating data of both surveys was high (r = 0.985, *p* < .001, see Fig 1). The rating scale data were analyzed with cumulative link mixed effects models using the *ordinal* package (version 2022.11–23; [37]) in R (version 4.2.2). The factor *survey* (Survey 1 target-prime vs. survey 2 prime-target) was coded as 0.5 and -0.5, and the factor *condition* was coded using deviation coding with the unrelated condition as baseline (strongly vs. unrelated and weakly vs. unrelated). A model with a maximal random structure justified by the design was fitted: rating ~ survey * condition + (1 + condition | subject) + (1 + type | target). Results revealed significant differences between strongly related and unrelated word pairs (z = 36.70, *p* < .0001) and between weakly related and unrelated word pairs (z = 32.20, *p* < .0001). There was no main effect of survey and no interactions between survey and condition (all zs < 1). A posthoc t-test using the package *emmeans* (version 1.8.3) revealed that also the ratings for weakly and strongly related word pairs were significantly different (z = -32.71, *p* < .0001).

**Table 3:** Mean ratings and standard deviations (in parentheses) for the three conditions in Survey 1 and Survey 2.

Condition	Survey 1 (Target-Prime)	Survey 2 (Prime-Target)	Mean of Survey 1 and Survey 2
Strongly related	5.70 (0.80)	5.75 (0.84)	5.73 (0.82)
Weakly related	4.13 (1.16)	4.15 (1.19)	4.14 (1.17)
Unrelated	1.25 (0.25)	1.30 (0.23)	1.27 (0.25)

### Discussion

Rating data revealed that strongly related word pairs were indeed rated highly semantically related and unrelated word pairs were rated as very unrelated. The weakly related word pairs were rated significantly lower than strongly related word pairs, and importantly they were rated significantly higher than the unrelated word pairs. Both surveys resulted in remarkably similar semantic relatedness ratings for the three conditions. Thus, the order in which the words of each pair were presented in the surveys did not affect the ratings. The ratings for the word pairs obtained in this study were used to select the best set of stimuli for Experiment 2 that involved a semantic priming lexical decision task.

## Experiment 2: Semantic priming with the lexical decision task

### Methods

#### Participants

Seventy-two native speakers of Polish (57 female; *M* age = 24, SD = 1.40; 50 right-handed) participated in the study. They were first year students in the bachelor program at the Faculty of English, Adam Mickiewicz University in Poznań and they received course credits for their participation.

#### Materials

Stimuli were selected based on the results of Experiment 1. From the 264 triplets, those rated above 4 were included in the strongly related condition, word pairs rated above 3 and below 5.5 were included in the weakly related condition, and word pairs with scores below 2 were included in the unrelated condition. In total, 48 triplets were removed following the above criteria, which amounted to 216 word pairs in each of the three conditions.

Ratings obtained in Experiment 1 revealed that for 7 prime-target pairs the weakly related primes were rated as more related than the strongly related primes. Therefore, the weakly related primes were replaced with strongly related primes in these 7 word pairs. Furthermore, in 7 cases the unrelated word pairs scored above or slightly below 2. These primes were replaced with prime words selected from the list of previously rejected words, creating new unrelated pairs.

The new unrelated pairs were evaluated by seventy-one students at the Faculty of English, Adam Mickiewicz University in Poznań (34 participants evaluated the pairs in the ‘target-prime’ order and 37 participants evaluated the pairs in the ‘prime-target’ order to ensure that the order did not influence the results). All 7 exchanged unrelated pairs were evaluated below 2. The final set contained 216 prime-target pairs across three conditions (strongly related, weakly related, and unrelated word pairs). The characteristics of prime and target words and pairs are presented in Table 4.

**Table 4:** Prime and target word characteristics of 216 word pair triplets: Length, word frequency (Zipf values), and the semantic relatedness values (cosine similarity scores) for the strongly related, weakly related, and unrelated conditions tested in Experiment 2.

Characteristics	Mean	SD (min.—max.)
Target word frequency	3.6	0.4 (2.9–4.1)
Target word length	6.3	1.4 (4–9)
***Prime word frequency***		
strongly related	2.9	0.8 (1.1–5.5)
weakly related	3.2	0.8 (1.3–5.3)
unrelated	3.5	0.4 (2.9–4.7)
***Prime word length***		
strongly related	7.2	2.2 (3–15)
weakly related	6.4	1.8 (3–12)
unrelated	6.7	1.5 (4–9)
***Semantic relatedness values***		
strongly related	0.69	.07 (0.48–0.86)
weakly related	0.50	.05 (0.42–0.75)
unrelated	0.12	.13 (-0.18–0.51)

For the lexical decision task, a set of pseudowords was generated with Pseudo (https://waltervanheuven.net/pseudo/index.html) using 300 words taken from SUBTLEX-PL [30] that were selected using the following criteria: Zipf value (*M* = 4.68, range = 4.37–5.96) and word length in letters (*M* = 6.49, range = 5–11). For each word, five pseudowords were generated by replacing a random letter in the word by a letter of the same category (vowel or consonant) and checking that the pseudoword was not an existing Polish or English word. Furthermore, the program also checked that the pseudoword contained only valid Polish bigrams and trigrams. From this initial list, a final list of 144 pseudowords was selected by 5 independent judges. The judges were native speakers of Polish and they evaluated the pronounceability of the pseudowords.

#### Design

A within-subjects design was used for two sets of target words. The independent variable was the semantic priming condition: strongly related, weakly related, and unrelated. The dependent variables were reaction times and accuracy. In half of the trials the prime, e.g., *skała* (*rock*), was followed by a pseudoword, e.g., *UŚLIUCH*.

Target words were divided into two separate sets and for each set three lists were created so that each target word was preceded by each of its primes across lists. This procedure resulted in six lists, none of which was repeated within participants because each participant saw only one list. Each list included 36 strongly related, 36 weakly related, and 36 unrelated word pairs as well 36 semantically unrelated filler word pairs to ensure that half of the materials were related word pairs, and the other half, unrelated word pairs. The filler word pairs were the same in each list. Furthermore, 144 pseudowords were added to each list. Each pseudoword was preceded by a word and none of these real words appeared as either prime or target in the set of experimental materials. Thus, each participant completed 288 trials in total, of which 108 trials were experimental trials representing the three conditions of interest. Participants were randomly assigned to lists. Each list was completed by 12 participants (similar sample size as in [17]).

#### Procedure

The study was conducted in dimly lit cabins in the Language and Communication Laboratory at the Faculty of English at Adam Mickiewicz University in Poznań using E-Prime 2.0. First, participants read the information about the study, signed a written informed consent form, and completed the handedness questionnaire [38]. Participants were randomly assigned to one of six sets in the study. In the practice session, participants had a chance to familiarize themselves with the procedure and needed to obtain a minimum of 80% correct responses to be admitted to the main experiment. The practice session could be repeated a maximum of 3 times for each participant. None of the participants was excluded from the study based on the results of the practice session. After the practice session, participants proceeded to the experimental block.

Each trial started with a fixation cross (+) presented for 500 ms, followed by a prime presented in lower case for 150 ms, followed by a blank screen presented for 50 ms, and finally the target word was presented in upper case for a maximum of 1,000 ms. Stimuli were presented using the font Calibri (size 32) in black on a gray background. Participants performed a lexical decision task, in which they were instructed to press the right Enter key when the target word was an existing Polish word and the left Control key when the target word was not an existing Polish word. The assignment of response keys was counterbalanced across participants.

### Results

**Fig 2:**
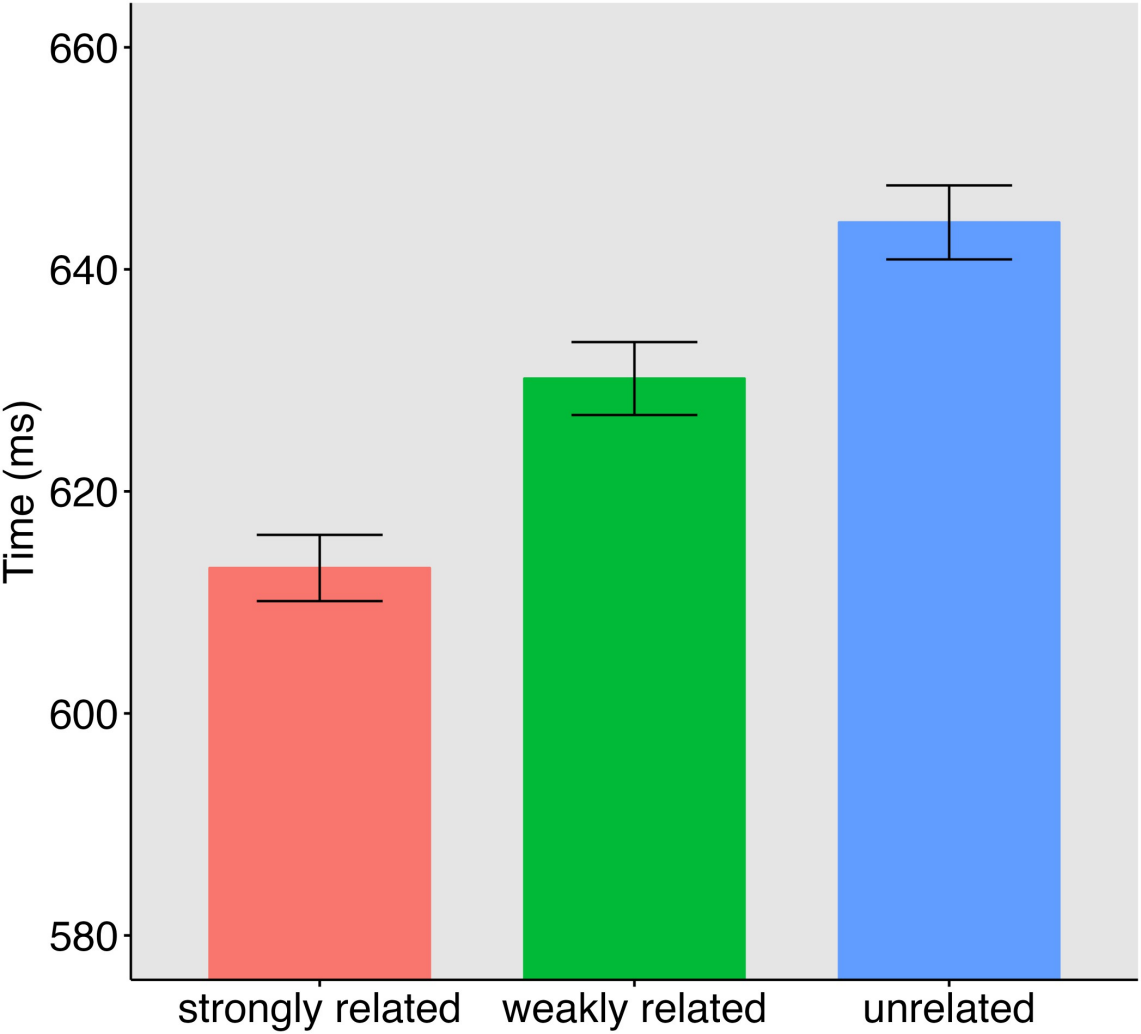
Mean reaction times and standard errors for the three priming conditions in Experiment 2.

The average time participants needed to complete the task was *M* = 11min and 18s (*SD* = 0.07). Overall error rate was very low (2.2%) and only correct response time data were analyzed. Reaction times slower or faster than 2.5 x standard deviation of the participant and item mean in a condition were considered outliers, as well as reaction times below 250 ms. Overall, less than 1% of the data were considered outliers. Data were analyzed with linear mixed effects models using the lme4 package (version 1.1–29) in R (version 4.2.1). Means and standard deviations are presented in Table 5 and Fig 2. To reduce skewness of the reaction times, an inverse transformation was applied. Deviation coding was used for *condition* (unrelated, weakly related, strongly related) with unrelated as the baseline, and the between subject factor *set* was coded as -.5 and.5. Modeling started with a full model with the maximal random structure. This model did not converge, therefore the random structure was simplified until the model converged. The final model was specified as invRT ~ set * condition + (1 | subject) + (1 + condition | target). Relative to unrelated primes, a significant priming effect was found for weakly related primes (14 ms; t = -4.17, *p* < .001) and strongly related primes (31 ms; t = -8.17, *p* < .001). There was no effect of group (t < 1), nor interactions between group and condition (t’s < 1). A paired t-test also revealed a significant difference between weakly and strongly related primes (17 ms; t = 4.05, *p* < .001). Furthermore, an orthogonal polynomial contrasts analysis with emmeans revealed a significant linear contrast for condition (*z* = -8.17, p < .001).

**Table 5:** Mean reaction times in milliseconds with standard deviations (in parentheses) and error rates for the priming conditions in each set and both sets combined in Experiment 2.

Condition	Set 1		Set 2		Combined	
	RTs	Errors	RTs	Errors	RTs	Errors
Closely related	608 (148)	1.2 (0.31)	618 (152)	1.2 (0.30)	613 (150)	1.2 (0.21)
Weakly related	630 (175)	1.1 (0.29)	630 (156)	1.2 (0.30)	630 (166)	1.1 (0.21)
Unrelated	644 (177)	1.8 (0.37)	645 (157)	1.3 (0.32)	644 (168)	1.5 (0.24)

### Discussion

The results of Experiment 2 are clear and in line with the similarity ratings obtained in Experiment 1. Significant semantic priming effects were obtained with strongly related and weakly related primes in the lexical decision task. Importantly, response times for the three conditions showed a linear pattern with fastest responses observed for the strongly related condition, slowest responses for the unrelated condition, and with the RTs for the weakly related condition falling in-between. As a consequence, priming effects for strongly related primes were about two times stronger than those for weakly related primes (31 vs. 14 ms). This finding is consistent with other studies using the same task [2,7].

The observed semantic priming effects are likely automatic in nature. Similar experimental procedures, with short (below 250 ms) Stimulus Onset Asynchronies (SOAs), have previously been shown to tap automatic processes [12,17,39]. Furthermore, several studies have demonstrated that the manipulation of the relatedness proportion (the proportion of related word pairs in the materials) does not modulate these effects [40,41], which further supports the idea that procedures like the one employed in Experiment 2 elicit automatic priming effects.

In the current study, the Pearson correlation between the semantic relatedness ratings of Experiment 1 and the reaction times of Experiment 2 is -0.20 (*p* < .001). This is slightly stronger than the correlation of -0.18 (*p* < .001) between the vector similarity values of the semantic space used for stimulus selection (nkjp_wiki_forms_all_300_cbow_ns, [11]). In the next section, we will explore how other semantic spaces can predict our behavioral data.

## Experiment 3: Predicting behavioral data with semantic vectors

Previous research has found that semantic spaces are better predictors of reaction times than human ratings [10]. It is possible that the specific semantic space used for stimulus selection was not optimal and that other semantic spaces might outperform the human relatedness ratings in terms of predicting the response times in the semantic priming lexical decision task. In this section, we will explore this by evaluating existing and new Polish semantic spaces in a nested cross-validation setup that allows to reduce the risk of overfitting and to estimate the performance of the selected models on new datasets.

### Methods

#### The semantic vectors

We evaluated the 106 spaces published within the CoDeS project (Mykowiecka, Marciniak [11]; retrieved from http://dsmodels.nlp.ipipan.waw.pl/). We also trained a set of new vectors using a Polish corpus that included:

- a Polish subset of the c4corpus [42] (about 1.24 billion tokens), from which we removed all pages from Wikipedia that we were able to identify
- the subtitle corpus that was created for calculating the Polish subtitle frequencies [30] (about 146 million tokens)
- the dump of the Polish Wikipedia (238 million tokens)

To balance the corpus during the training, the order of documents originating from these sources was randomly shuffled. We also created an alternative version of the corpus by processing it using the spaCy Python module (the *pl_core_news_sm* model) in which each inflected word form was substituted by its lemma.

We trained the *word2vec* semantic vectors using the gensim package [43]. Because the training procedure, in addition to the selection of the corpus, also requires a number of hyperparameters, we explored this hyperparameter space by training a substantial number (763) of the semantic spaces. The set of hyperparameters was randomly chosen for each of the models from a prespecified set of choices (for a full list of parameters and possible values, see Table 6).

**Table 6:** List of hyperparameters and possible values, and hyperparameter values of the semantic space identified as optimal. For more information about the meaning of each hyperparameter see gensim documentation (https://radimrehurek.com/gensim/models/word2vec.html#gensim.models.word2vec.Word2Vec).

Hyperparameter	Candidate values	Value
**corpora**	form-based or lemmatized corpus	lemmatized corpus
**size**	100, 200, 300, 400, 500, 600, 800, 1000	400
**alpha**	0.001, 0.005, 0.02, 0.025, 0.03, 0.05, 0.1	0.05
**window**	3, 5, 6, 7, 8, 9, 10, 11, 12, 15	8
**min_count**	30, 50, 100, 200, 300	50
**sample**	0.0, 0.0001, 0.001, 0.01, 0.1	0.001
**min_alpha**	0.0001, 0.0005, 0.001, 0.005	0.001
**sg**	0, 1	0
**hs**	0, 1	0
**negative**	5, 10, 15, 20	15
**cbow_mean**	0, 1	1
**iter**	1, 2, 3, 5, 7	1

#### Model selection procedure

In order to compare different models and select the optimal ones, we used the semantic priming dataset from Experiment 2, the ratings reported in Experiment 1 and the SimLex-999 dataset (similarity and relatedness ratings [44]).

Finding a model that generalizes well to datasets other than the relatively small evaluation datasets that we have available is among the main goals of the current paper. We also wanted to ensure that we correctly estimated the performance of different models on new datasets. To achieve these goals we employed nested cross-validation (k*l-fold cross validation (28))–a resampling method that assesses how the results would generalize to an independent dataset.

The main intuition behind this procedure is captured in the division of the full dataset into three subsets: a training dataset, a validation dataset, and a test dataset. Such a division reflects the three stages that are necessary to get to a good model and also obtain a prediction of how it would generalize to new datasets: 1) training statistical models with various predictors (in this case relatedness measures derived from various semantic spaces), 2) selecting a model that performs best among the candidates using the validation dataset, and finally 3) measuring the performance of the selected model on a test dataset on which the model was not trained or selected. The performance of the model on the test dataset provides an estimate of its performance on new datasets (drawn from the same probability distribution as the test dataset).

Depending on how the dataset is split into the three subsets, the outcome of the procedure may differ. To correct for this, the nested cross validation procedure can be applied. This approach boils down to repeatedly splitting the full dataset into such three datasets to make the cross-validation procedure more exhaustive, which is especially important when the size of the evaluation datasets is limited.

In our evaluation procedure, we used a 5-fold validation in the outer loop and a 5-fold validation in the inner loop of the nested cross validation procedure. The outer loop involved dividing the full dataset into subsets and then the *k-1* subsets were used to select the best model while the remaining (test) subset was used to determine how well the model is expected to generalize to independent datasets. The inner loop was used to select the best model in terms of the predictors included and the optimal semantic space. The inner loop operated on the training dataset created by subsetting the full dataset in the outer loop. It then further divided that training set into *l* subsets where *l-1* subsets were used to train the linear regression model with different sets of predictors and the remaining subset was used to calculate the expected performance of the models. The model that performed best was subsequently passed to the outer loop for evaluation. This setup ensured that the choice of the optimal semantic space was treated as another layer of parameter estimation.

We considered four tasks in the model selection and testing: predicting the LDT log RTs, ratings from the current study, and similarity and relatedness ratings from SimLex-999. In the case of predicting LDT log RTs, we fitted linear regression models with various predictors to evaluate the amount of variance in the log RTs that could be accounted for. Predictors included word frequency (Zipf values as reported in SUBTLEX-PL; [30]) and word length (number of letters) of both the prime and the target word. The range for these values was limited in the stimulus selection procedure, which limits the potential variance explained by these predictors. We then fitted various models that, in addition to the baseline predictors, included a measure of semantic distance between the prime and the target estimated by various semantic spaces and human ratings collected as part of the current study. We have fitted the models using a subset of data and looked at how much variance was explained in the test set. We used the pairs of stimuli from all conditions (strongly related, weakly related and unrelated word pairs).

We followed an equivalent procedure for prime-target relatedness ratings from the current study (an average from Survey 1 and Survey 2), and similarity and relatedness from the SimLex-999 dataset. In the case of these behavioral measures, there were no additional predictors included in the model beyond the semantic similarity measures.

The model selection was done by measuring performance (variance explained) in each of the four tasks independently and then taking the model with the lowest average rank across these tasks as the best model.

### Results and discussion

We estimated the likely lower-bound on the performance of the selected models by averaging the variance explained by the models selected in all five iterations of the outer loop. The resulting averages are presented in Table 7.

**Table 7:** The predictiveness of the linear models using the optimized semantic model hyperparameters. These performance numbers can be considered to represent the estimated performance of the models on the independent datasets.

Datasets	R^2	n
***LDT / log RT***	0.1317	648
***Relatedness ratings (current study)***	0.7468	648
***Relatedness ratings (SimLex-999)***	0.3930	999
***Similarity ratings (SimLex-999)***	0.1585	999

In order to establish how the predictions of LDT log RTs based on the semantic vectors would compare to human ratings (average from Survey 1 and Survey 2), we also included them in the analysis for predicting the log RTs. The amount of variance explained by human ratings was 0.1194, much lower than well performing semantic models.

**Fig 3:**
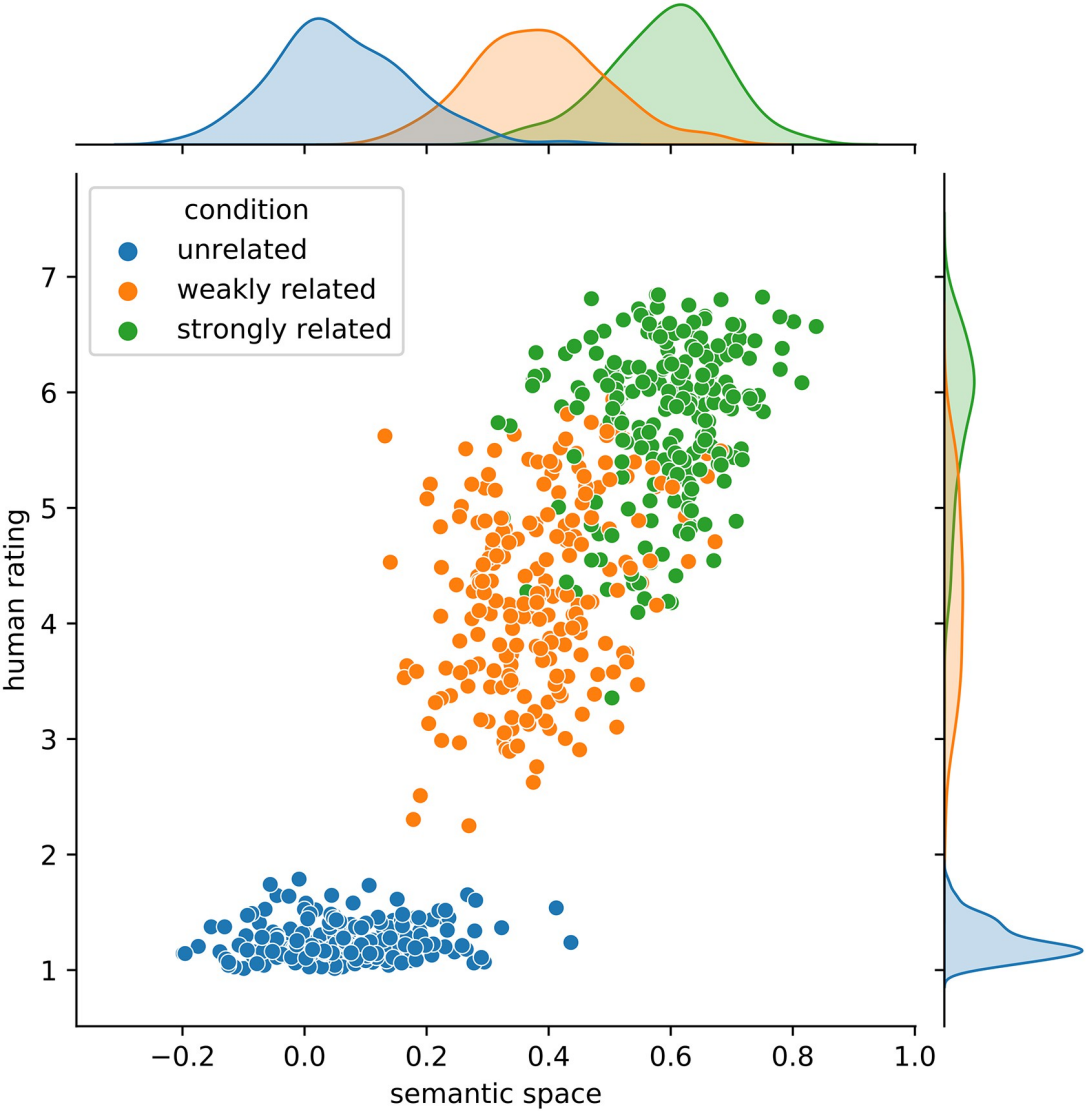
Scatterplot showing the relationship between the semantic space similarity estimates (*nkjp+wiki-lemmas-restricted-300-cbow-ns*.*txt*.*gz*, [11]) and the human similarity ratings.

The above procedure based on nested-cross validation provides an indication of the predicted performance on independent datasets, but it does not clearly identify the space that should be shared with other researchers as in principle each iteration of the outer loop may be concerned with a different semantic space. To identify the final semantic space, we ran one last iteration of the inner loop, which trains and selects the best model, on the full dataset. This procedure identified one of the spaces published as part of the CoDeS project (*nkjp+wiki-lemmas-restricted-300-cbow-ns*.*txt*.*gz*; the relationship between the relatedness estimates in that space with human relatedness rating is depicted in Fig 3) as the optimal one. Among the spaces trained as part of the current study, the CBOW model with the hyperparameters reported in Table 6 turned out to perform best. Additionally, models were ranked based on all four datasets (ratings, RTs, SimLex-rel, and SimLex-sim), only the behavioral dataset obtained in Experiment 1 and Experiment 2 (ratings and RTs), and only SimLex (SimLex-rel and SimLex-sim). The model that was optimal for all datasets (*nkjp+wiki-lemmas-restricted-300-cbow-ns*.*txt*.*gz)*, was also optimal for the behavioral dataset of Experiment 2, and was second in rank when only SimLex-999 was used (for details and the full list of models see Supplementary Materials).

Finally, we tried to illustrate why following the procedure of fitting the model on each dataset separately and then reporting the *R^2* from the same dataset that the model was trained on, without splitting it into train and test set, can be misleading with small datasets. In order to do that, we fitted the models to predict LDT log RTs using the baseline predictors and the semantic spaces that we trained. Out of the models that were created as part of the current study, the model that gave the best performance in such a procedure had an *R^2 = 0*.*1478* on the training dataset. However, when we trained the linear model using the semantic space with the same hyperparameters on a train set and then estimated variance explained on the independent test dataset it appeared to be much lower (*R^2 = 0*.*1316*). Moreover, this semantic space turned out to perform badly on other datasets (*R^2 = 0*.*2569* for human ratings, in SimLex-999 *R^2 = 0*.*1266* for the relatedness ratings, and *R^2 = 0*.*0069* for the similarity ratings).

## General discussion

The main aims of the current study were to 1) develop a well-tested dataset of word pairs in Polish that represent strong and weak semantic relations, and 2) to select a semantic model that can be used by other researchers to predict human performance in various tasks. We used the nested cross-validation method to test whether the model would be sufficiently robust for a range of materials.

The aim of Experiment 1 was to test whether the word pairs selected based on one of the semantic spaces published by Mykowiecka, Marciniak [11] (nkjp_wiki_forms_all_300_cbow_ns) were indeed perceived as representing strong and weak semantic relations by human participants. It is important to note that the words were preselected by two human judges from a pool of 800 word pairs, and this intermediate step contributed to the selection of the best word pairs from the pool. The analysis of the data obtained in Survey 1 and Survey 2 revealed that the word pairs selected based on semantic vectors were evaluated as expected based on semantic similarity values. Also, a strong positive correlation was found between the results of the two surveys, which means that the order of prime and target word presentation did not modulate the ratings.

The aim of Experiment 2 was to test a carefully controlled subset of the word pairs used in Experiment 1 in a semantic priming lexical decision task (LDT). The results revealed a graded effect in the reaction times, with shorter reaction times for weakly related than unrelated primes, but longer than for strongly related primes. This result is in line with previous studies on indirect semantic relations or moderate associations [2,7,8], and shows that the strength of the semantic relation between the prime and target modulates lexical decision latencies to target words. Importantly, the current study used a semantic priming LDT, whereas most studies that investigated indirect semantic relations used a semantic judgment task, which suggests that these semantic effects are task independent. Further research can examine this aspect more directly and systematically, for example, by implementing within-participants designs.

The model selection procedure used in Experiment 3 allowed us to identify a semantic space that can be expected to perform well across several behavioral tasks. By applying the nested cross-validation procedure, we also provided realistic estimates of how well it may perform on independent datasets. The additional analysis, in which we compared the expected performance of the semantic space selected using this procedure, shows that, even though we decided to setup a selection procedure that attempts to find a compromise between performance on different tasks, the resulting semantic space can still perform better than human ratings when used to predict priming LDT RTs.

Furthermore, we have demonstrated that, especially when only small datasets are available, a selection procedure that is based on a single task and that involves using only performance scores from the training dataset can be misleading. First, it leads to an overly optimistic estimate of the performance on the independent dataset. Secondly, it easily overfits and may lead to selecting a semantic space that generalizes poorly to different datasets (both for the same and different tasks).

The model that was optimal (*mykowiecka*.*nkjp+wiki-lemmas-restricted-300-cbow-ns*.*txt*.*gz*) for our behavioral datasets and the SimLex-999 Polish datasets was based on lemmas from the National Corpus of Polish and Wikipedia. In contrast, the model originally used for material selection in the current study (*nkjp+wiki-forms-all-300-cbow-ns*) was based on word forms rather than lemmas. Overall, it seems that models based on lemmas do better than those based on word forms for our behavioral dataset, although stimulus material included only nouns in the nominal case. Marciniak, Mykowieck [44] also reported that lemma-based models performed better than those based on word forms for the SimLex-999 relatedness and similarity scores. Polish is a morphological complex language and therefore future work could explore the use of morph-fitting to improve the semantic quality of the vector spaces [45].

Future research may build on the fact that applying the nested cross-validation method enables comparing performance not just of the same models with different parameter sets, but even completely different types of models. Previous research evaluating psycholinguistic resources (word frequencies, semantic similarity measures) assumed that the relationship between various behavioral measures and their predictors is best represented by linear regression models [10]. Applying nested cross-validation in such studies makes it easy to compare different types of models that may not share this assumption.

Finally, we would like to point out one limitation of the applied method. It estimates the performance of the models on independent datasets, but it is important to keep in mind that it assumes that the datasets would need to be drawn from the same probability distribution. This is likely not the case given that there is a large variation in stimuli selection for various studies originating from vastly different needs for answering different research questions.

Overall, the present paper presents a well-matched database of word pairs in Polish that vary in the degree of semantic relatedness, which can be used by other researchers in studies on semantic processing. The materials were first selected using semantic vectors, then tested in two studies that involved the collection of data from participants, and finally various semantic models were used to examine which models are optimal in predicting the human data. The outcomes of this study that relate to semantic models clearly corroborate previous findings [10] in that they show very strong correlations between human ratings and semantic vectors, as well as predict semantic priming effects obtained in behavioral reaction times studies better than human ratings. This means that future research in language processing can utilize semantic models rather than rating data from surveys, which will potentially ease the material selection process.
